# Isotonic quadriceps endurance is better associated with daily physical activity than quadriceps strength and power in COPD: an international multicentre cross-sectional trial

**DOI:** 10.1038/s41598-021-90758-7

**Published:** 2021-06-02

**Authors:** Erik Frykholm, Sarah Gephine, Didier Saey, Arthur Lemson, Peter Klijn, Eline bij de Vaate, François Maltais, Hieronymus van Hees, André Nyberg

**Affiliations:** 1 Department of Community Medicine and Rehabilitation, Physiotherapy, Umeå University , Umeå, Sweden; 2 Institut universitaire de cardiologie et de pneumologie de Québec, Université Laval, Quebec, Canada; 3 Univ. Lille, Univ. Artois, Univ. Littoral Côte D’opale, ULR 7369-Urepsss, Lille, France; 4 Department of Pulmonary Diseases, Radboud UMC, Nijmegen, The Netherlands; 5 Department of Pulmonary Rehabilitation, Merem Medical Rehabilitation, Hilversum, The Netherlands; 6 Department of Pulmonary Medicine, Amsterdam University Medical Center, Amsterdam, The Netherlands

**Keywords:** Chronic obstructive pulmonary disease, Rehabilitation

## Abstract

Knowledge about modifiable determinants of daily physical activity (PA) in patients with chronic obstructive pulmonary disease (COPD) is crucial to design effective PA interventions. The present study aimed to determine the contribution of quadriceps strength, power and endurance to daily PA in COPD. Additionally, for quadriceps endurance, we also aimed to determine to what extent the association varies according to the mode of movement (isotonic, isometric, or isokinetic). Using a multicentre cross-sectional trial design we determined the contribution of quadriceps function to daily PA (steps, sedentary time and time spent doing moderate-to-very-vigorous physical activity [MVPA]) using bivariate and partial Pearson correlation analysis (r) and multiple linear regression models (ΔR^2^). Pre-determined controlling factors were sex, age, body mass index (BMI), COPD-assessment test, forced expiratory volume in one second in percent of the predicted value (FEV_1pred_), and distance walked on the 6-minute walk test. Eighty-one patients with COPD (mean ± SD: age 67 ± 8 years, FEV_1pred_ 57 ± 19%, daily steps 4968 ± 3319, daily sedentary time 1016 ± 305 min, and MVPA time 83 ± 45 min) were included. Small to moderate bivariate correlations (r = .225 to .452, *p* < .05) were found between quadriceps function and measures of PA. The best multiple linear regression models explained 38–49% of the variance in the data. Isotonic endurance was the only muscle contributor that improved all PA models; daily steps (ΔR^2^ = .04 [relative improvement 13%] *p* = .026), daily sedentary time (ΔR^2^ = .07 [23%], *p* = .005) and MVPA-minutes (ΔR^2^ = .08 [20%], *p* = .001). Isotonic endurance was also independently associated with most PA variables, even when controlling for strength, power or isometric-isokinetic endurance properties of the muscle (r = .246 to .384, *p* < .05). In contrast, neither strength, power, isometric-or isokinetic endurance properties of the muscle was independently associated with PA measures when controlling for isotonic endurance (r = .037 to .219, *p* > .05). To conclude, strength, power, and endurance properties of the quadriceps were low to moderately associated with PA in patients with COPD. Isotonic quadriceps endurance was the only quadriceps property that was independently associated with the different measures of PA after controlling for a basic set of known determinants of PA, quadriceps strength or power, or isometric or isokinetic quadriceps endurance. Future longitudinal studies should investigate its potential as a modifiable determinant of PA.

## Introduction

Physical activity (PA) is recognized as an essential feature in patients with chronic obstructive pulmonary disease (COPD)^1^, being amongst the strongest predictors of all-cause mortality and quality of life in the disease^2–5^. Importantly, PA levels can potentially be improved^1^, and over recent years, several modifiable determinants of PA in COPD have been identified^3^. Even so, there is still limited and inconsistent evidence on the effectiveness of interventions to enhance PA in COPD^1^. Considering that knowledge about determinants of PA in patients with COPD is crucial to design effective PA interventions^3,6^, these findings highlighting that other modifiable determinants of PA likely exists, even though they have yet to be identified^1,3^.

Quadriceps dysfunction, defined by reduced strength, power or endurance properties of the quadriceps is a common extrapulmonary manifestation of the disease, evident in over a third of the COPD-population^7,8^. Even though improving quadriceps function often is an integrated aim of exercise interventions^1,7,9–11^, information on the potential role of quadriceps function as a determinant of PA in COPD is sparse.

Other than quadriceps strength, shown not to be linked to PA in several different studies^12–14^, there is today limited knowledge on the impact of other potentially modifiable properties of the quadriceps on PA in COPD. Recently, quadriceps power has emerged as a more important predictor of PA than quadriceps strength, being positively associated with active time per day as well as light intensity PA in COPD^13^. However, to our knowledge, the role of quadriceps power to PA has only been investigated in this single study in which the sample size was small, and the study included only older men, highlighting a need for further investigation^13^.

Another potentially modifiable determinant of PA is quadriceps endurance. The endurance capacity of the quadriceps may be of particular interest to PA, considering that the muscle's ability to repeat a specific task over time^15^ indicates a similarity in PA task requirements based on the repetitive nature of quadriceps use in everyday daily activities such as walking. For example, quadriceps endurance has previously been found to be independently associated with walking distance on the 6-minute walk test (6MWT) even after controlling for quadriceps strength^16^. Considering the existing link between 6MWT and PA in COPD^3,17^, these findings indicate that quadriceps endurance could also be more closely associated with PA than other aspects of quadriceps function.

The primary aim of the present study was, therefore, to determine and to compare the contribution of quadriceps strength, power and endurance to PA in patients with COPD. Furthermore, in recent studies, static (isometric) and dynamic (isotonic, isokinetic) endurance protocols have been found to reflect different aspects of quadriceps function in COPD^16,18^. Thus, a secondary objective was to determine to what extent the association varies according to the mode of movement (isotonic, isometric, isokinetic). Amongst the quadriceps muscle properties examined, the main hypothesis was that quadriceps muscle endurance would show the strongest association and contribution to the physical activity measures.

## Method

### Study design and participants

The present study was a multicentre cross-sectional study conducted in Sweden, Canada, and the Netherlands. Participants in this prospective study were continuously enrolled with convince sampling at each centre. Participants were included in every month of the year, thereby covering all seasons. Inclusion criteria were at least 40 years of age, and a confirmed diagnosis of mild to very severe COPD according to the Global Initiative for Chronic Obstructive Lung Disease (GOLD) criteria^19^ controlled through medical records. Exclusion criteria’s consisted of recent COPD exacerbation (**within 3 months**), muscular-, rheumatic-, or cardiac disorders affecting testing procedures, and a daily dose of prednisone >10mg within 3 months, also controlled through medical records, and self-reported regular quadriceps strengthening exercises.

### Procedure

**Figure 1:**
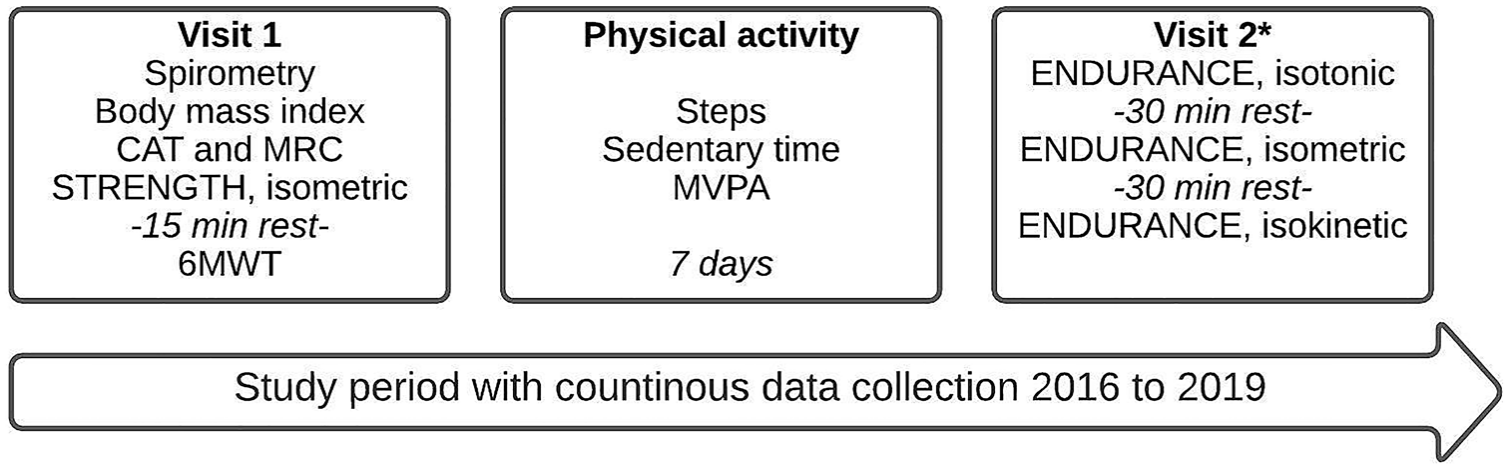
Description of study procedure. *CAT* COPD assessment test, *MRC* Medical Research Council dyspnea scale, *6MWT* 6-minute walking test, MVPA time spent in moderate to very vigorous intensity. *Randomized order, POWER, was obtained from the isokinetic protocol.

All participants attended two visits, and PA was measured for seven days, from 2016 to 2019. On the first visit were anthropometrics including body mass index (BMI), and symptoms on the COPD assessment test (CAT) collected. After that were a 6MWT^20^, spirometry^21^, and an isometric quadriceps strength test performed. The second visit included the assessment of isokinetic quadriceps power as well as isometric, isokinetic and isotonic quadriceps endurance^22^ (Fig. 1).

#### Quadriceps function

Quadriceps strength, power and endurance were measured with a computerized dynamometer (Biodex System Pro 3 or 4 Biodex Medical Systems, Shirley, New York, NY, USA) using test procedures that have been described in detail elsewhere^22^. Quadriceps strength was measured during a maximal voluntary contraction (MVC) using an isometric (static) protocol at a 90° knee angle^22^, performed in line with international recommendations and reported in Newton-meters (Nm)^7^. Quadriceps power, equivalent to energy output per unit of time, was obtained from the average power developed during 30 maximal isokinetic contractions; results were expressed in watt^23,24^.

Quadriceps endurance was assessed with three reliable protocols^22^, all performed with similar (1) starting position (sitting, 90° knee angle, straps over the thigh and pelvis), (2) direction of movement (restricted by the lever arm), and (3) duration of the test (~ 60 s). The three protocols differed regarding the mode of contraction/movement (isometric [static], isokinetic [dynamic, fixed speed], or isotonic [dynamic, fixed external loading]). The two dynamic protocols had identical range-of-motion (90° knee flexion to full individual knee extension − 5°), while the static protocol was performed at a 90° knee angle^22^. Standardized verbal encouragements were given in all tests.

The isometric protocol consisted of a static contraction at 60% of MVC at 90° knee angle using visual feedback (results: total work (load [Nm] × seconds until momentary failure)^22^. The isokinetic endurance protocol consisted of 30 maximal dynamic contractions at a fixed pace of 90°/s (results: total work in Joule [J])^22^. The isotonic endurance test protocol consisted of the maximal amount of dynamic leg extensions throughout the full range of motion, performed using a resistance of 30% of isometric MVC maintaining an externally set pace by a metronome at 60 beats per minute (i.e., 30 repetitions/minute) (results: total work (load [Nm] × repetitions)^22^.

#### PA

A tri-axial accelerometer (DynaPort MoveMonitor, McRoberts; Hague, the Netherlands) was used to measure PA. The accelerometer is considered valid for patients with COPD^25^ and is a small, light ambulant measurement device (106.6 x 58 x 11.5 mm, 55 g) worn with an elastic belt on the lower back. Since the sensors measure gravity (*g*) in static situations, the acceleration signal is expressed relative to *g*. The participants were instructed to wear the accelerometer for seven consecutive days, including a weekend day. The minimum wearing criteria was ≥ 4 days, with ≥ 8 h wearing time each day^26,27^. Daily steps, daily sedentary time in minutes, and daily time spent in moderate to very vigorous activity (MVPA) were selected as these are the three most commonly used measures of PA in COPD intervention studies^1^ and were considered to display different aspects of PA. PA measures were summarized for valid days by the manufacturer software. Thresholds for describing time spent at different intensity levels were; sedentary time < 1.5 metabolic equivalents (METs), and time spent in moderate to very vigorous activity ≥ 3 METs, as proposed by the American College of Sports Medicine^28^. To define each individual’s average value during the physical activity measurement was the median from valid days used in the main analysis. To compare levels of PA between week- and weekend days, the individual’s median value during the valid week and weekend days was used.

### Statistical analysis

A statistical software package was used for all calculations (Jamovi, version 1.6.7, Sydney, Australia), a *p* value of < .05 was considered to indicate statistical significance. Contribution of quadriceps function to PA (daily steps, daily sedentary time, and daily MVPA-minutes) was determined through Pearson’s bivariate and partial correlations, and multiple linear regression analyses. Levels of PA between week- and weekend was compared using paired t-tests and Pearson’s bivariate correlations. For bivariate analysis were correlation coefficients (r) values in the range of < .1 considered trivial, .1–.3 small, .3–.5 moderate, .5–.7 large, .7–.9 very large, and > .9 extremely large^29^. Partial correlations analyses were used to examine the correlation between PA and one quadriceps property while controlling for another quadriceps property (e.g., the correlation between MVPA-minutes and quadriceps endurance while controlling for quadriceps strength). In the multiple linear regression analysis, daily steps, daily sedentary time, and daily MVPA-minutes represented dependant variables. Based on previous literature^3,13^ and basic clinical characteristics^7^ were sex, age in years, BMI, CAT score, FEV_1pred_ and 6MWT in meters selected as pre-determined independent variables and constituted the basic model^30,31^. A minimum of 75 participants was aimed to be included based on the general rule that 10 to 15 subjects per predictor are needed for a reliable equation^32^. To determine the contribution of various quadriceps properties to PA in COPD, isometric strength, isokinetic power, as well as isotonic, isometric and isokinetic quadriceps endurance measurements were entered independently to the basic model. The models were then again applied to the three dependent PA variables. Outcomes to evaluate the contribution to PA included the standardized estimated effect of included variables, the predicting ability of the model using R^2^, and absolute change (ΔR^2^) between the first and second models. Multicollinearity was evaluated with the tolerance and variance inflation factor (VIF) statistics with tolerance value > .1 and VIF < 10. The quality of the linear regression models was assessed using the Akaike information criterion (AIC) (lower score, better model)^33^.

### Ethics approval and consent to participate

The study was conducted in accordance with the declaration of Helsinki and was approved by the respective local ethical boards (regional medical ethics committee of Umeå: DNR: 2015-426-31M, 2016-379-32M; Comité éthique du Centre de Recherche, Institut Universitaire de Cardiologie et de Pneumologie de Québec, Université Laval: CER: 21322; Committee on Research Involving Human Subjects (CMO) region Arnhem-Nijmegen: CMO: NL59926.091.16), all participants received written and verbal information about the study and gave their written informed consent before the study commenced.

## Results

Eighty-one participants with COPD were included in the analysis, 4 participants were excluded from the analysis, either missing a valid isometric quadriceps muscle endurance measurement (n = 3) or isotonic quadriceps muscle endurance measurement (n = 1). The PA measurement covered both weekdays and weekends for all participants. Eighty participants had at least one valid weekend day. Table 1 display participant characteristics, PA measures, and quadriceps functions, Table 2 displays bivariate and partial correlation coefficients, Table 3 displays the R^2^, and ΔR^2^ of each multiple linear regression model, while Table 4 displays the standardized estimated effect of the models with the best coefficient of determination for daily steps, sedentary time, and MVPA-minutes. No multicollinearity was observed in the multiple analyses.

**Table 1:** Participant characteristics.

Characteristic	n = 81
Age, years	67 ± 8
Sex, females/males	35/46 (43/57)
FEV_1_, L	1.5 ± .6
FEV_1pred_, %	57 ± 19
FVC, L	3.3 ± .9
FVC_pred_, %	98 ± 21
FEV_1_/FVC, %	46 ± 13
GOLD grade, n I/2/3/4 (%)	12/37/23/9 (15/46/28/11)
MRC, n 1/2/3/4/5 (%)	1/35/15/8/22 (1/43/19/10/27)
CAT, 0–40	16 ± 7
BMI, kg/m^2^	27 ± 5
6MWT, m	452 ± 123
6MWT % predicted	78 ± 23
**Physical activity**	
Wearing time, %	83 ± 20
Valid days, n	7 ± 1
Steps, n	4968 ± 3319
Sedentary time, minutes	1016 ± 305
Moderate to very vigorous activity, minutes	83 ± 45
**Quadriceps function**	
STRENGTH, MVC, Nm	132 ± 47
STRENGTH, MVC % predicted	73 ± 18
POWER, Watt	59 ± 23
ENDURANCE, isotonic, Nm × repetitions	1274 ± 1065
ENDURANCE, isometric, Nm × seconds	3842 ± 2325
ENDURANCE, isokinetic, Joule	2001 ± 862

Values are means ± standard deviations or number and (%). Activity measures are means of individual daily medians of minutes or steps, of at least four days of measurements and at least eight hours of wearing time.

Thresholds for describing time spent at different intensity levels are proposed by the American College of Sports Medicine: Sedentary time < 1.5METs, time spent in moderate to very vigorous activty ≥ 3 to > 9 METs.

*FEV1* forced expiratory volume in the first second, *FEV1pred* FEV1 in percent of predicted, *FVC* forced vital capacity, *FVCpred* FVC in percent of predicted, *GOLD* Global Initiative for Lung Disease, *MRC* Medical Research Council dyspnea scale, *CAT* COPD assessment test higher values indicating more symptoms, *BMI* body mass index, *6MWT* 6-minute walk test, *MVC* maximal isometric voluntary contraction, *METs* metabolic equivalents.

**Table 2:** Pearson bivariate and partial correlation (r) analysis coefficients between quadriceps functions and daily physical activity measures .

	Steps per day	Sedentary time	MVPA-minutes
**Quadriceps function**			
STRENGTH, MVC	0.264*	− 0.259*	0.353**
+ controlling for POWER	0.060	− 0.124	0.095
+ controlling for ENDURANCE, isotonic	0.037	− 0.062	0.066
+ controlling for ENDURANCE, isometric	0.068	− 0.163	0.148
+ controlling for ENDURANCE, isokinetic	0.067	− 0.118	0.115
POWER, Watt	0.313**	− 0.347**	0.409***
+ controlling for MVC	0.184	− 0.239*	0.239*
+ controlling for ENDURANCE, isotonic	0.128	− 0.101	0.177
+ controlling for ENDURANCE, isometric	0.132	− 0.273	0.223*
+ controlling for ENDURANCE, isokinetic	0.047	− 0.338**	0.098
ENDURANCE, isotonic, Nm × Reps	0.346**	− 0.435***	0.452***
+ controlling for MVC	0.246*	− 0.366***	0.307**
+ controlling for POWER	0.201	− 0.279*	0.272*
+ controlling for ENDURANCE, isometric	0.174	− 0.384***	0.279*
+ controlling for ENDURANCE, isokinetic	0.200	− 0.365***	0.280*
ENDURANCE, isometric, Nm × seconds	0.367***	− 0.225*	0.422***
+ controlling for MVC	0.273*	− 0.096	0.268*
+ controlling for POWER	0.241*	− 0.258*	0.249*
+ controlling for ENDURANCE, isotonic	0.217	− 0.040	0.219
+ controlling for ENDURANCE, isokinetic	0.242*	− 0.100	0.258*
ENDURANCE, isokinetic, Nm	0.315**	− 0.256*	0.402***
+ controlling for MVC	0.191	− 0.110	0.233*
+ controlling for POWER	0.061	− 0.003	0.047
+ controlling for ENDURANCE, isotonic	0.134	− 0.025	0.169
+ controlling for ENDURANCE, isometric	0.140	− 0.160	0.217

*MVC* maximal isometric voluntary contraction, *MVPA* time spent in moderate to very vigorous intensity.

*p < .05, **p < .01, ***p < .001.

**Table 3:** Multiple linear regression analyses.

Model	R^2^	ΔR^2^	*p*	AIC
**Steps per day**				
Basic model	.373			1520
+ STRENGTH, MVC	0.376	<0 .01	0.559	1522
+ POWER, Watt	0.381	0.01	0.356	1521
+ ENDURANCE, isotonic	0.415	0.04	0.026*	1517
+ ENDURANCE, isometric	0.400	0.03	0.053	1518
+ ENDURANCE, isokinetic	0.387	0.01	0.214	1521
**Sedentary time**				
Basic model	0.309			1805
+ STRENGTH, MVC	0.310	< 0.01	0.722	1807
+ POWER, Watt	0.332	0.02	0.114	1804
+ ENDURANCE, isotonic	0.379	0.07	0.005**	1798
+ ENDURANCE, isometric	0.316	< 0.01	0.367	1806
+ ENDURANCE, isokinetic	0.312	< 0.01	0.551	1807
**MVPA-minutes**				
Basic model	0.407			1482
+ STRENGTH, MVC	0.422	0.01	0.184	1482
+ POWER, Watt	0.432	0.02	0.082	1480
+ ENDURANCE, isotonic	0.489	0.08	0.001**	1472
+ ENDURANCE, isometric	0.446	0.06	0.006*	1476
+ ENDURANCE, isokinetic	0.439	0.03	0.048*	1480

The basic model includes; sex, age in years, body mass index, COPD Assessment Test score, forced expiratory volume in the first secondin % of predicted, and 6-minute walk test distance.

*ΔR*^*2*^ change in R^2^ from the basic model. *AIC* Akaike information criterion, *MVC* maximal isometric voluntary contraction, *MVPA* time spent in moderate to very vigorous intensity.

*p < .05, **p < .01, ***p < .001.

**Table 4:** Standardized estimates of the multiple linear regression models with the best coefficient of determination for sedentary time, daily steps, and MVPA-minutes.

	Daily steps				Daily sedentary time				MVPA-minutes			
	*p*	Stand. estimate	95% CI		*p*	Stand. estimate	95% CI		*p*	Stand. estimate	95% CI	
			Lower	Upper			Lower	Upper			Lower	Upper
**Basic model**												
Sex	0.94	0.01	− 0.19	0.21	0.21	0.13	− 0.08	0.34	0.68	0.04	− 0.15	0.23
Age	0.42	− 0.08	− 0.28	0.12	0.60	0.05	− 0.15	0.26	0.17	− 0.13	− 0.31	0.06
BMI	0.08	− 0.18	− 0.38	0.02	0.95	0.01	− 0.20	0.23	0.01*	− 0.25	− 0.44	− 0.06
CAT	0.29	0.11	− 0.10	0.31	0.12	0.17	− 0.05	0.38	0.61	− 0.07	− 0.25	0.10
FEV_1pred_	0.02*	0.27	0.05	0.49	0.33	− 0.11	− 0.11	0.33	0.07	0.19	− 0.03	0.30
6MWT	< 0.01**	0.35	0.11	0.60	< 0.01**	− 0.37	− 0.63	− 0.12	< 0.01**	0.32	0.09	0.55
+ ENDURANCE, isotonic†	0.03*	0.25	0.03	0.48	< 0.01**	− 0.33	− 0.56	− 0.10	< 0.01**	0.36	0.15	0.56

Models with a measure of quadriceps endurance are shown when there was a significant change in the coefficient of determination from the basic model including; gender, age in years, BMI, CAT, FEV1pred and 6MWT.

*MVPA* time spent in moderate to very vigorous intensity, *BMI* body mass index, *CAT* COPD assessment test, higher values indicating more symptoms, *FEV1pred* forced expiratory volume in the first second in percent of predicted, *6MWT* 6-minute walk test.

*p < .05, **p < .01, ***p < .001, ^†^the best quadriceps function from the multiple linear regression analyses.

### Pearson’s correlation and multiple linear regression analyses

Small to moderate significant correlations (r = .219 to .452) were found between different quadriceps function properties and all PA measures. Partial correlation analyses on strength, power and endurance properties of the quadriceps revealed that isotonic quadriceps endurance was independently associated with all PA measures after controlling for quadriceps strength as well as independently associated with sedentary time and MVPA-minutes but not daily steps (r = .201, *p* = .07) when controlling for quadriceps power. In contrast, neither strength nor quadriceps power was independently associated with PA measures when controlling for isotonic endurance. Similarly, other than daily steps when controlling for isokinetic endurance (r = .200, *p* = .08), isotonic quadriceps endurance was also independently associated with PA measures when controlling for isometric and isokinetic endurance. At the same time, no significant associations were seen between PA measures and isometric and isokinetic endurance when controlling for isotonic endurance (Table 2).

The basic multiple linear regression model, including the pre-determined variables sex, age in years, BMI, CAT score, FEV_1pred_ and distance walked on the 6MWT explained 31–41% of the variance in the PA data (R^2^: daily steps .37, daily sedentary time .31 and MVPA-minutes .41). Isotonic quadriceps endurance was included in the best model to additionally explain variability of daily steps (ΔR^2^ = .04 [relative improvement of the model = 13%] *p* = .026), daily sedentary time (ΔR^2^ = .07 [23%], *p* = .005) and MVPA-minutes (ΔR^2^ = .08 [20%], *p* = .001), resulting in 38–49% of the variance in PA being explained (Tables 3 and 4). Quadriceps strength or power did not improve any model while isometric endurance improved the MVPA model (ΔR^2^ = .06 [10%], *p* = .006). The MVPA model also improved by isokinetic endurance (ΔR^2^ = .03 [8%], *p* = .048) (Table 3).

Group means of the individual’s average value using medians for valid week- and weekend days separately show higher daily steps and daily MVPA-minutes during weekdays than during weekends (mean ± standard deviation: weekday daily steps 5162 ± 3498 vs. weekend daily steps 4685 ± 3203, *p* = .029, weekday MVPA-minutes 86.6 ± 48.5 vs. weekend MVPA-minutes 80.8 ± 45.0, *p* = .040), sedentary time was not different (weekday sedentary minutes 1014 ± 300 vs. weekend sedentary minutes 1015 ± 302, *p* = .993). The correlations between weekday and weekend PA were very large (r = .84–.87, *p* < .001).

## Discussion

The main findings of the present study are that strength, power and endurance properties of the quadriceps are significantly associated with PA in patients with COPD. Of the included measures of quadriceps function, quadriceps endurance was the only muscle property that was associated with daily steps, daily sedentary time and MVPA-minutes even after controlling for a basic set of known determinants of PA or when controlling for strength-power properties of the muscle. Our result also indicate that the mode of movement is of importance as isotonic quadriceps endurance was independently associated with PA in COPD even when controlling for isometric or isokinetic quadriceps endurance. In contrast, neither strength, power nor isometric and isokinetic endurance properties were independently associated with PA measures, when controlling for isotonic endurance.

### Interpretation of study results

Quadriceps strength was associated with all PA measures, however, when added to a basic set of known determinants of PA (sex, age, BMI, FEV_1pred_, CAT, 6MWT) quadriceps strength was not a significant contributor to any of the models. These findings are in line with previous research^12,13,34^. However, considering the known link between BMI and quadriceps strength in COPD^35^, it should be noted that the lack of added predictive value of quadriceps strength to the basic set of variables, may, at least partly, be explained by that BMI was one of the basic variables. Furthermore, quadriceps power was recently found to be positively associated with light intensity PA, and active time per day (r = .37 to .51), but not daily steps in older men with COPD (r = .30, *p* > .05)^13^. In the present study, active time per day and light intensity PA was not explicitly investigated but isokinetic quadriceps power was associated with all included aspects of PA, even daily steps (r = .313, *p* < .01). About the latter, considering the similarity in the coefficient of correlation in the present study (r = .313) and in the study of Hernandez et al. (r = .300), the lack of statistical significance in the latter study was likely due to the lower sample size (n = 44 vs. n = 81)^13^. Nevertheless, even though quadriceps power has been highlighted as a more important predictor of PA than maximum strength^13^, the results from the present study indicate that it does not seem to add additional value when added to already known determinants of PA in COPD, or when compared to the endurance capacity of the quadriceps. It should be noted that the recognised link between isokinetic power and distance walked on the 6MWT^18^ likely contributed to why the basic models were not further improved by isokinetic power. However, in contrast, the endurance capacity of the quadriceps improved PA models when added to the basic models despite the known link to distance walked on the 6MWT^16,36^. Our observed findings are further supported by the results from the partial correlation analysis in which neither quadriceps strength nor power was independently associated with PA when controlling for isotonic quadriceps endurance. In contrast, isotonic quadriceps endurance was, except for steps per day when controlling for quadriceps power, independently associated with daily steps, sedentary time and MVPA-minutes even after controlling for strength and power properties of the quadriceps. Similarly, isotonic quadriceps endurance was independently associated with most PA measures even after controlling for isometric or isokinetic quadriceps endurance measurements.

### Clinical relevance

As previously reported, FEV_1pred_, BMI, and distance walked on the 6MWT were significant contributors to the best models to determine PA. In fact, the distance walked on the 6MWT is consistently found associated with measures of PA in COPD, and often considered one of the best available determinants of PA in COPD^3^. The findings from the present study demonstrate that strength, power and endurance properties of the quadriceps are also low to moderately associated with PA in patients with COPD. Isotonic quadriceps endurance was the only significant muscle contributor to all PA models, resulting in an absolute improvement of the basic models with 4 to 8% (relative improvement 13 to 23%). Isotonic quadriceps endurance was also independently associated with PA in COPD even after controlling for strength/power or other modes (isometric/isokinetic). In contrast, neither strength/power nor other modes of quadriceps endurance was associated with any of the included PA measures when controlling for isotonic endurance. A possible explanation for these findings is that isotonic endurance measurements, in a similar way as many daily physical activities, e.g., going for a brief daily walk, consists of dynamic submaximal movements/contractions. In contrast, isometric measurements are static (i.e., no movement) while isokinetic power and endurance measurements consist of maximal, and not submaximal contractions^8,22–24^.

The relevance of the association of isotonic quadriceps endurance when estimating PA can be extrapolated to a clinical example. Based on the predictive models, an improvement of roughly 37% in isotonic quadriceps endurance would be needed to achieve a clinically relevant 600-daily step improvement in PA^37^, if all other factors remain constant. For the 6MWT, the corresponding change for a similar improvement in isotonic quadriceps endurance based on our data would be 63 m. Even though these estimations should be interpreted cautiously due to the cross-sectional nature of the data which prevent concluding on cause and effect, other research groups have found that trials with significant improvement in PA have also demonstrated considerable improvements in distance walked on the 6MWT (47 to 79 m)^38–40^. One of the studies also reports improvements of isometric quadriceps endurance of ~ 35% and isometric quadriceps strength of ~ 14% after an intervention with high-intensity interval training on stationary bicycles with significant improvements in several PA measures^40^. To our knowledge, isotonic quadriceps endurance has not been evaluated in trials that have demonstrated significant improvements in PA. Still, isotonic quadriceps endurance is repeatedly shown to be highly adaptable (+ 50 to 97%) by exercise interventions^11,41,42^.

Taken together with the observation that isotonic endurance also was independently associated with most PA measures even when controlling for strength, power, or isometric- and isokinetic endurance properties of the quadriceps, our results indicate that isotonic quadriceps endurance seems to be more closely associated with PA than other aspects of quadriceps function. Nevertheless, and as further described in the strengths and limitations of the study, only about half or less of the variation in PA is explained in our models, despite including a wide range of known determinants of PA among patients with COPD, and bivariate and partial correlations mainly demonstrated low to moderate associations for quadriceps muscle properties and PA in COPD.

## Strengths and limitations

The international multicentre design with continuous data collection, the broad spectrum of disease severity and the direct comparison of several different aspects of quadriceps function are key strengths of the current study, increasing generalizability and facilitating the interpretation of the role of quadriceps function to PA in COPD. However, in line with the findings from the present study, it should be noted that strength, power and endurance properties of the quadriceps could be measured in various ways^8,43–45^. Thus, we cannot be certain that the results would have been similar if other strategies were also used. Nevertheless, isometric MVC was chosen as a measure of quadriceps strength since it, to date, is the recommended approach to assess quadriceps strength in COPD^7^. For quadriceps power, no such specific assessment recommendation exist^44^. However, other strategies such as the stair climb power test^46^, sit to stand^47^ or isotonic measures of power^24^ exist and may have provided other information, even though this needs to be further investigated. Furthermore, even though several different PA measures exist^1,3^, daily steps, sedentary time and MVPA-minutes were selected as these are the three most commonly used outcomes of PA in intervention studies targeting PA in COPD^1^. Steps and MVPA-minutes were lower during the weekend than during weekdays (− 476 steps/day and − 5.8 MVPA-minutes), but with very large correlations, as shown previously in COPD^48^. Inclusion of both week and weekend days resulted in more valid days in the main analysis, and the associations, therefore, reflect an average of valid days and not any specific part of the week. Lastly, an important notion is that only about half or less of the variation in PA is explained in our models, despite including known determinants of PA among patients with COPD^3^. The explanatory degree in the present study may have been further improved by the inclusion of more known factors than those selected^3,49,50^.

Firstly, have limitations in chest wall volume measurements, and central hemodynamic and peripheral muscle oxygenation been investigated and show that quadriceps muscle oxygen saturation and inspiratory reserve chest wall volume as the significant best contributors, explaining 77.7% of the variance in daily movement intensity^49,50^. However, movement intensity was not used as a dependent variable in this study, and movement intensity and movement amount (steps and time in activity) might reflect different aspects of PA^6,48^.

Secondly, in combination with the notion that included participants, even though representative in all GOLD stages, had a relatively well-preserved functional exercise capacity (only 22% walked < 350 m on the 6MWT). Factors other than pure physiological factors, such as motivation, social participation, meaningful interpersonal interactions, readiness to change and self-efficacy may have provided additional value and should be considered in future studies^1,51^. And thirdly, testing also other muscle groups, such as the calf-muscles, could also provide additional value considering the know link between the calf-muscles and walking ability in COPD^52^. Nevertheless, we do believe that this basic set of factors was sufficient for the present study's aim, that is, to determine and compare the association between different aspects of quadriceps function and PA in COPD.

## Conclusion

In conclusion, this study found that strength, power and endurance properties of the quadriceps were low to moderately associated with PA in patients with COPD, but that isotonic quadriceps endurance was the best, and the only quadriceps property that was associated with all the different measures of PA (daily steps, daily sedentary time and MVPA-minutes) even after controlling for a basic set of known determinants of PA or when controlling for strength or power properties of the muscle. Thus, altogether these findings highlight that the endurance capacity of the quadriceps seems to be a new potentially modifiable determinant of PA in COPD that may be considered if the goal is to optimize the role of exercise training as a mean to improve PA in COPD, even though this needs to be confirmed in future longitudinal studies. Furthermore, when examining quadriceps endurance, the mode of movement seems to be important as isotonic quadriceps endurance was independently associated with most PA measures even when controlling for isometric and isokinetic endurance properties of the quadriceps.

## Data Availability

The datasets used and/or analyzed during the current study are available from the corresponding author on reasonable request.
